# Does an Audio Wearable Lead to Agitation Reduction in Dementia: The Memesto AWARD Proof-of-Principle Clinical Research Study

**DOI:** 10.21203/rs.3.rs-6008628/v1

**Published:** 2025-02-17

**Authors:** Raj Shah, Santosh Basapur, Kirsten Hendrickson, Julie Anderson, Jamie Plenge, Amelia Troutman, Eukesh Ranjit, Jeff Banker

**Affiliations:** Rush University Medical Center; Rush University Medical Center; Rush University Medical Center; Edgewater Safety Systems, Inc; Rush University Medical Center; Rush University Medical Center; Southern Illinois University School of Medicine; Edgewater Safety Systems, Inc

**Keywords:** Alzheimer’s Disease and Related Dementias, agitation, device, residential care facilities

## Abstract

**Background:**

Agitation is a common behavioral symptom in persons living with Alzheimer’s disease and Alzheimer’s disease-related dementias (AD/ADRD), especially in the setting of residential care. Pharmacologic and non-pharmacologic interventions are limited. Memesto is a wearable audio device designed to provide messages and music that can be scheduled or played on demand. The objective of this proof-of-principle study was to quantify whether Memesto can reduce agitation in persons with AD/ADRD.

**Methods:**

Persons living with AD/ADRD with a Clinical Global Impressions-Severity (CGI-S) average score of 4 or greater, one informal caregiver, and one formal caregiver (triad) were recruited from residential care facilities in the Midwest region of the United States. After consent and a two-week training period, the triad was monitored every two weeks from Baseline to Week 10 with the Neuropsychiatric Inventory (NPI) agitation domain subscale (primary endpoint) and the CGI-S scale (secondary endpoint) with the last observation carried forward. The average score on the NPI agitation domain subscale and the CGI-S scale at Baseline and Week 10 as rated by the two caregivers were compared. A 30% drop in the NPI agitation domain subscale in 50% of the persons living with AD/ADRD was considered a clinically meaningful finding.

**Results:**

Over thirteen months of recruitment, 9 triads were identified in 6 residential care facilities in three Midwestern states. For the NPI agitation domain, 6 of 9 (67%) persons with AD/ADRD had a 30% reduction in the average caregiver ratings at Week 10. No adverse events were identified associated with the use of the device. Device usability was rated as positive based on a survey.

**Discussion:**

This study provided quantitative data on psychometrically sound agitation scales regarding a 10-week treatment course with Memesto after a two-week training period. The results were limited by the inability to recruit the desired set of 20 triads due to disruptions in care and staff at residential care facilities. Further effectiveness testing in a larger cohort with a sham control device is necessary.

**Trial Registration:**

www.clinicaltrials.gov. NCT05153161. First posted December 10, 2021.

## INTRODUCTION

Alzheimer’s disease and Alzheimer’s disease-related dementias (AD/ADRD) represent the most common forms of dementia, with an estimated 4.9 to 6.9 million Americans living with AD/ADRD.^1,2^ AD/ADRD are age-related neurodegenerative diseases characterized by losses in memory, orientation, independent decision-making capacity, and self-care. In residential care settings in the United States, the prevalence of agitation (behavioral disruptions such as aggression, combativeness, shouting, exit-seeking, and disinhibition) has been reported to be 50%.^3^ Lack of effective care approaches and technologies for treating agitation leads to poor quality of life for persons living with AD/ADRD and their caregivers and increases health care utilizaion.^4,5^

A means of detecting agitation as it increases and automatically delivering an effective, calming intervention could have benefits. Based on studies on the effectiveness of simulated presence therapy, repetitive messaging, and reminiscence therapy,^6,7^ Edgewater Safety Systems, Inc., developed a smart media player (Memesto) worn by the person living with AD/ADRD that delivers personalized music and pre-recorded messages. The device was informally piloted on AD/ADRD persons in residential care facilities. The majority of the eleven caregivers involved stated that the device was useful in lowering agitation.

The objective of this proof-of-principle study was to generate quantitative data for the continued development of the Memesto platform as a caregiver-engaged audio therapy intervention to reduce agitation in persons with AD/ADRD living in residential care facilities.

## METHODS

### Study Design and Hypotheses.

The study design consisted of a single open-label treatment arm. The primary hypothesis is that treatment with Memesto will result in a reduction in the average 10-week Neuropsychiatric Inventory^8^ (NPI) agitation domain score compared to the average Baseline score. The secondary hypothesis is that treatment with Memesto will result in a reduction in the average 10-week Clinical Global Impressions Scale-Severity^9^ (CGI-S) score compared to the average Baseline score.

### Ethics and Regulatory Approval.

The Rush University Medical Center Institutional Review Board (IRB) approved the study. Written documentation of informed consent was obtained from each member of the triad. Since this study was designed to involve persons with moderate to severe AD/ADRD who have exhibited agitation while residing in residential care facilities, it was anticipated that most of these persons would not have the capacity to consent at the time of enrollment; therefore, the power of attorney/authorized decision maker for the person living with AD/ADRD was approached to provide signed consent for his or her engagement in the study.

### Setting.

The study was conducted by the Rush Alzheimer’s Disease Center at Rush University Medical Center, Chicago, Illinois, USA. Using its relationships with residential care facilities that participated in prior National Institute on Health-funded longitudinal cohort studies, Rush staff reached out to contacts in the Chicagoland region regarding participation in the AWARD pilot study. Additional facilities were added through relationships established by the site Principal Investigator (PI) with geriatricians and other residential care leadership, including those geriatricians in the Division of Geriatric and Palliative Medicine at Southern Illinois University School of Medicine in Springfield, Illinois. Also, sites involved in prior work on the qualitative assessment of Memesto in southwest Michigan and northeast Indiana were approached. After an initial warm hand-off discussion with the facility’s AD/ADRD care leadership, an in-person or virtual meeting was arranged to share more information about the study and to determine the feasibility and interest of the facility participating.

### Participants:

Participants were adults with AD/ADRD who had clinically significant agitation, defined as a state of poorly organized and purposeless psychomotor activity including at least one of the types of agitation: aggressive verbal (screaming, cursing), aggressive physical (destroying objects, grabbing), or non-aggressive physical (restlessness, pacing) behaviors. Inclusion and exclusion criteria are shown in Box 1. Screen failures were defined as participants who consented to participate in this study but did not proceed into the 10-week treatment phase.

Each participant had two study partners: a family caregiver (informal study partner) and a professional caregiver from the residential care facility (formal study partner) willing to participate in the study with him or her. Each facility shared the IRB-approved flyer with families of persons living with AD/ADRD and agitation. Written materials on dementia, agitation, and Memesto were made available. Research study staff then engaged individually with each potential participant and informal study partner who expressed interest in the study. All study procedures, risks and benefits, and the option to withdraw without penalty were reviewed. After all parties had as much time as needed to consider participation, signed consents were obtained, and copies were provided for their records.

Study retention was supported by having a two-week training period built into the protocol. Also, study staff maintained close follow-up with the triad. A participant was considered lost to follow-up if he or she repeatedly failed to participate in scheduled visits and could not be contacted by study staff after at least 3 documented attempts. All participants who permanently discontinued study treatment and did not agree to continued data collection through study completion (Week 10) were withdrawn from the study.

#### Study Device

The Memesto device existed as an off-the-shelf, commercially available wi-fi-enabled cellular phone coupled with a mobile operating system- and web-based application that enables recording and loading of audio media via the internet. The device was not used as a cellular phone and did not have cellular phone service. A neck lanyard with a waterproof case was provided with the device to facilitate use and accountability. In addition, based on feedback from residential care facility leaders, options to have a wristwatch wearable, Bluetooth-enabled speaker, and noise-cancelling headphones were also provided as needed. After completing the two-week training phase, participants were given the device and continued using it for 10 weeks of bi-weekly data collection. Edgewater Safety Systems, Inc. staff was available throughout the study for device- or application-related questions or troubleshooting.

#### Study Activities

Study activities by visit type over the 12 weeks of engagement are diagrammed in Table 1. After obtaining informed consent, the screening visit was completed to collect participant demographic and medical history information to determine eligibility. All participants meeting eligibility criteria (apart from a demonstration of the use of the device) were trained on how to use the Memesto platform, and training was reinforced over the two-week training phase. Upon satisfactorily completing this phase, a Baseline visit was conducted to administer scales for the primary and secondary agitation outcomes, concomitant medication use, including antipsychotic medication use, adverse events, and administration of study-related surveys and questionnaires. The Baseline assessments were repeated every 2 weeks (Weeks 2, 4, 6, 8, and 10) either in-person or via telephone. The end of the study was defined as the completion of the Week 10 visit or Early Termination (ET) visit.

#### Data Collection

Data collection was the responsibility of trained study staff under the delegation and supervision of the site PI. Clinical data was entered directly from source documents into REDCap, a 21 CFR Part 11-compliant data capture system.^10,11^

### Outcome Variables.

The primary study endpoint was the NPI agitation domain subscale, with a secondary study endpoint of the CGI-S scale; both were assessed as the average change from Baseline to the end of treatment (Week 10). The NPI is a widely used, well-established instrument that assesses behavioral changes in neurologic illnesses through a structured interview with a caregiver.^8^ Behavioral domains are scored based on frequency and severity. The nursing home version of the inventory was utilized in this study and administered (in-person or via telephone) to both the formal and informal study partners by qualified and trained study staff.^12^ The CGI-S is a brief observer-rated instrument that establishes a global rating of illness severity.^9^ The scale responses range from 1 (normal) to 7 (amongst the most extremely ill) with a score of zero assigned if the item was not assessed. The CGI-S related to agitation was administered (in-person or via telephone) to both the formal and informal study partners by qualified and trained study staff.

The site PI and study staff monitored safety via adverse event reporting. Adverse event reporting began at Screening and continued until the end of the study. The occurrence of adverse events was sought by non-directive questioning of the participant and/or study partners during each study visit or volunteered through unsolicited contact by the participant and/or study partners between study visits. Given this study was anticipated to be minimal risk, only adverse events associated with use of the Memesto device or with study-related questioning were recorded.

The Post-Study System Usability Questionnaire (PSSUQ) is a 16‐item standardized questionnaire that was used to measure the participants’ and study partners’ perceived satisfaction with the intervention.^13^ The responses to each PSSUQ statement have a range of integer scores from 1 to 7, where 1 is the highest rating (“strongly agree”) and 7 is the lowest rating (“strongly disagree”). An option of not applicable was also provided.

#### Statistical Methods

The population for treatment and safety analyses was participants who consented and completed the Baseline visit. The general analytic approach was to compare outcome scores at the Week 10 visit with the Baseline scores. Missing data was handled by the last observation carried forward method. As this research was designed as a proof-of-principle study, no formal power calculation was conducted.

Participant baseline demographics (such as age, gender, race/ethnicity) and health characteristics (such as Baseline scores on agitation measures) were summarized using numbers and percentages for categorical variables and by means, medians, standard deviations, and ranges for continuous variables.

The pre-specified quantitative endpoint was changed from Baseline in the NPI agitation domain. The NPI agitation domain was rated by the two study partners for symptoms frequency (1: rarely – less than once per week, 2: sometimes – about once per week, 3: often – several times per week but less than every day, 4: very often – once or more times per day) and severity (1: mild – behavior is stressful for the resident but can be controlled by the caregiver, 2: moderate – behaviors are stressful for and upsetting to the resident and are difficult to control, 3: severe – agitation is very stressful or upsetting to the resident and is very di cult or impossible to control. There is a possibility they may injure themselves and medications are often required). A score of zero indicated no symptoms. The NPI’s agitation domain score is a composite (frequency × severity) score of 1 to 12. The NPI agitation domain score was determined from interviews with both study partners.

The average Week 10 NPI agitation domain score from the reports of the two study partners was compared to the Baseline average agitation score. A positive finding was that at least half of the participants had a 30% reduction in the NPI agitation domain score. A 30% decrease in scores is generally clinically meaningful.^14^ Therefore, a positive finding was that at least half of the participants would have a 30% improvement after 10 weeks of treatment.

A secondary endpoint was a validated agitation measure (change in the CGI-S score over the 10 weeks of data collection). The CGI-S was measured based on a 7-point Likert scale. The number of persons with a CGI-S score showing improvement was defined as the last observation carried forward score being three or less. A clinically meaningful signal was if improvement was noted in 30% of the participants.

Safety outcomes included adverse events related to the device or with study procedures. Device satisfaction was determined at the end of the study by calculating the median and maximum score on the PSSUQ for all study partners rating the device.

## RESULTS

### Participants.

**Figure 1:**
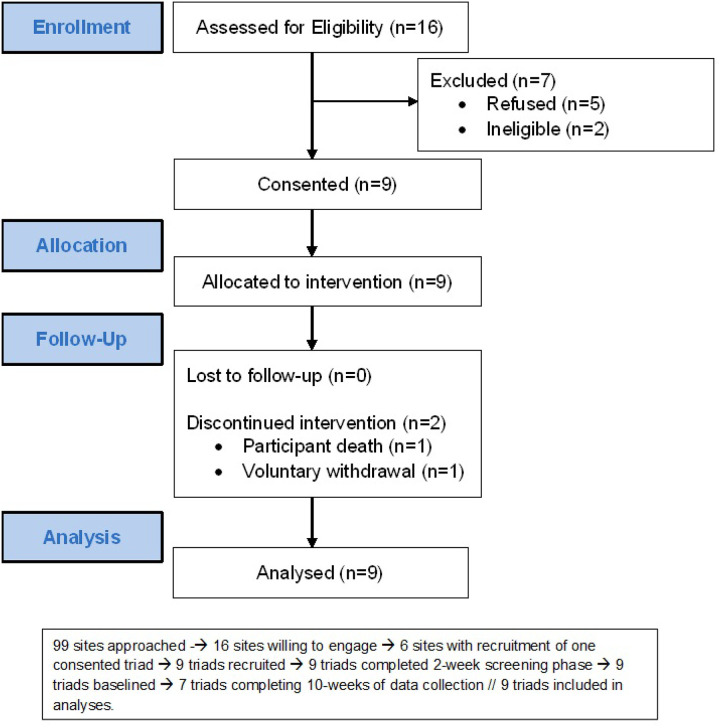
Participant flow diagram

Recruitment for the study started in August 2022 and continued until September 2023. It was significantly impacted by changing environments in residential care facility priorities and staffing due to the COVID-19 pandemic. The participant flow is shown in Fig. 1. Of the 9 triads that consented to participation, one triad exited voluntarily two weeks after the Baseline visit and went through an ET visit. No triad was lost to follow-up. One triad was not able to complete 10 weeks of follow-up due to the death of the participant between Week 8 and Week 10, but the values from their last visit were carried forward for analyses.

### Descriptive data.

The basic demographics of the participants and the two types of study partners are provided in Table 2. In brief, most participants were over the age of 75 years, were women, were non-Hispanic white, and had less than a college education. The mean CGI-S score at Screening for both the informal and formal study partners combined was 4.7 (+/− 0.3). The mean NPI agitation score at Baseline from both types of study partners was 5.7 (+/− 3.0). Informal study partners rated the NPI agitation score at Baseline slightly higher than the formal study partners (6.1 +/−2.8 vs. 5.3 +/−2.9, respectively); however, these were not statistically different (t(df = 16) = 0.6, p = 0.6). Informal study partners were mainly children of the participant, were college educated, and were non-Hispanic White. Most formal caregivers were middle-aged women, not college-educated, and non-Hispanic white. The formal study partner could act as the study partner for multiple participants at the facility.

### Ten-week change in NPI Agitation Subscale.

The average score on the NPI agitation domain from the reports of the two study partners at Week 10 was 3.0 (SD = 2.7). Informal study partners rated the NPI agitation domain score at the study end lower than the formal study partners (2.7 +/−2.4 vs. 4.1 +/−3.1, respectively); however, these were not statistically different (t(df = 16) = 0.6, p = 0.3). The average 10-week change in agitation domain score was − 2.7 (SD = 2.3). It was determined that 6 of 9 (67%) participants had a 30% reduction in the NPI agitation domain score, which is greater than the pre-defined clinically significant positive finding cut point of at least half of the participants would have a 30% improvement after 10 weeks of treatment.

### Ten-week change in CGI-S Scale.

The average CGI-S score from the reports of the two study partners at Week 10 was 3.3 (SD = 1.9). Informal study partners rated the CGI-S score at study end lower than the formal study partners (3.0 +/−1.8 vs. 3.7 +/−1.9, respectively); however, these were not statistically different (t(df = 16) = 0.6, p = 0.3). The average 10-week decline in the CGI-S score was 1.4 (SD = 1.5). It was determined that 4 of 9 participants (44%) had a 10-week CGI-S average score between the two study partners of 3 or less. Therefore, the pre-defined clinically significant positive finding cut-point was met: greater than 30% of the participants had an average CGI-S score of 3 or less at 10 weeks of treatment.

### Safety.

While there was a potential that using the device (e.g., wearing the device, listening to repeated music/voice messages) as well as answering study-related questions about the participant’s mood and behaviors may become upsetting, frustrating, and/or tiring to either the participant and/or study partners, these risks were not observed in the study. Study partners did not refuse study-related questions, and participation was not declined at any time due to the device.

### Device Satisfaction.

The number of respondents, median, and maximum scores are provided for each of the 16 questions by both study partners at the Week 10/ET visit (see Table 3). At Week 10/ET, 17 of the 18 study partners provided a response to the overall satisfaction with the device question, and the mean score was 2.0 (range 1 to 7); nine provided a “strongly agree” response and only one provided a “strongly disagree response.” Of the informal study partners, 4 out of 8 provided the “strongly agree” response to the overall satisfaction question; of the formal study partners, 5 out of 9 provided the highest satisfaction response.

## DISCUSSION

In this proof-of-concept study, designed to provide initial data to determine whether Memesto use quantitatively reduces agitation in persons with AD/ADRD, formal and informal study partners reported improvements in well-characterized agitation scales over a 10-week period. Study participation risks were minimal, and device satisfaction was high among the informal and formal study partners after 10 weeks of use.

While there are no similar quantitative study results from other devices in development using the same voice and music components, this study does show quantitative signals for improvement in agitation utilizing well-validated agitation rating scales with clinical benefit definitions. These same scales were used in clinical trials of pharmacologic treatments being developed for agitation in persons living with AD/ADRD. The use of conservative clinical cut points on validated agitation scales provided greater confidence in the findings of the pilot study. We maintained the high engagement of those participants by limiting the study burden and providing virtual data collection methods. We obtained user experience data to help facilitate continued improvements to the device.

The study was limited by not achieving the intended study cohort of 20 triads. This limited the robustness of the findings for the primary and secondary outcomes. Also, the diversity of participants was not achieved as hoped, although several additional sites than originally anticipated provided engagement in the Midwest United States. Finally, the open-label design has a risk of reporting bias. However, “triangulation” with ratings from two separate types of study partners was added to reduce some of the bias while still enabling a clear signal for further device development.

## CONCLUSIONS

This study provided quantitative data on psychometrically sound agitation scales regarding a 10-week treatment course with Memesto after a 2-week training phase. Further effectiveness testing in a larger cohort with a sham control device is necessary.

## Data Availability

The datasets used and/or analyzed during the current study are available from the corresponding author upon reasonable request. A request for data can be placed at the Rush Alzheimer’s Disease Center Resource Sharing Hub after review and approval (www.radc.rush.edu).
